# Lower Airway Infection Delaying the Diagnosis of Aortic Dissection in an Elderly Woman

**DOI:** 10.7759/cureus.20487

**Published:** 2021-12-17

**Authors:** Helena Luís, Bela Machado, Carolina Barros, Mariana Gomes, Mariana Bilreiro

**Affiliations:** 1 Internal Medicine, Hospital Central do Funchal - Serviço de Saúde da Região Autónoma da Madeira, Entidade Pública Empresarial da Região Autónoma da Madeira (SESARAM, EPERAM), Funchal, PRT; 2 Internal Medicine, Hospital Dr. Nélio Mendonça, Funchal, PRT

**Keywords:** diagnostic error, disclosure of medical error, clinic management, missed diagnosis, type a acute aortic dissection

## Abstract

Type A aortic dissection is a surgical emergency occurring when an intimal tear in the aorta creates a false lumen in the ascending aorta. The authors report the case of an older woman with a medical history of arterial hypertension, atrial fibrillation, dyslipidemia, heart failure, and osteoarticular spinal pathology, presenting with sudden and persistent retrosternal pain, who was initially misdiagnosed with a lower airway infection and was discovered to have an acute type A aortic dissection. The authors intend to draw attention to medical errors and emphasize the importance of early diagnosis in pathology with a potentially fatal prognosis.

## Introduction

Aortic dissection represents an uncommon, acute non-traumatic emergency of the aorta, with an incidence in the general population ranging from 2.6 to 3.5 per 100,000 person-years [1-4]. This condition results from a longitudinal detachment of the aortic intima and adventitia, leading to infiltration of blood to the medial layer [5]. However, if not recognized and treated promptly, it can be fatal [6].

The risk factors associated with this identity are hypertension (including that mediated by cocaine), atherosclerosis, prior cardiac or aortic surgery, bicuspid aortic valve, known aneurysm, and connective tissue disorder Marfan’s syndrome or Ehlers-Danlos syndrome [5,7-8]. In addition, aortic dissection is more common in men, and women tend to be older at presentation [8-9].

The extent of the dissection and the cardiovascular structures involved dictate the signs and symptoms of an acute aortic dissection. However, the most common presenting symptom is a sudden onset of chest pain, which patients describe as excruciating, anterior or interscapular that migrates along with the dissection [10]. Since acute chest pain can be present in other cardiovascular pathologies, as in acute coronary syndrome, this diagnosis can be easily forgotten so a high suspicion is required to obtain an early diagnosis, and the most appropriate medical or surgical treatment can be instituted promptly. Kurabayashi M et al. showed that a total of 109 patients with aortic dissection presenting to the emergency room (ER) had been misdiagnosed in 16% of cases, and this condition was not included in the initial differential diagnosis [11].

## Case presentation

An 82-year-old Caucasian female presented with sudden and persistent retrosternal pain and was sent to the ER by her relatives. The pain started the day before, and there was no radiation and no vomiting. She had a medical history of arterial hypertension, atrial fibrillation, dyslipidemia, heart failure, and osteoarticular spinal pathology. Upon observation, the patient was sweating, and initial vital signs were blood pressure of 83/57 mmHg, pulse of 90 per minute, respiratory rate of 27 per minute, temperature of 36.5ºC, and oxygen saturation of 94% breathing ambient air. No abnormalities were found on heart and lung examinations. The peripheral exam showed regular bilateral peripheral pulses. On the abdominal examination, the patient had a reducible umbilical hernia and a diffusely painful to deep palpitation.

Arterial blood gas revealed pH 7.45, partial pressure of carbon dioxide (pCO_2_) 36.7 mmHg, partial pressure of oxygen (pO_2_) 81.5 mmHg, oxygen saturation 94.2%, bicarbonate (HCO3-) 27.2 mEq/L, and lactates 1.3 mg/dL. Initial lab results showed a white blood cell count of 16.600 μL (normal range 4.200-10.800 μL), neutrophiles of 12.900 μL (normal range 1.900-7.200 μL), hemoglobin of 12.1 g/dL (normal range in women 12-16 g/dL), platelets of 285.000 μL (normal range 144.000-440.000 μL), creatinine of 0.71 (normal range 0.70-1.20 mg/dL), hyponatremia of 133 mEq/L (normal range 135-145 mEq/L), and hypokalemia of 3.3 (normal range 3.5-4.5 mEq/L), ALT of 11 U/L (normal range 14-54 U/L), aspartate aminotransferase (AST) of 20 U/L (normal range 10-35 U/L), LDH of 241 U/L (normal range <246 U/L), C-reactive protein (CRP) of 111.8 mg/L (normal range <6.1 mg/L), D-dimers of 5770 ng/mL (normal range <255 ng/mL), and troponin T of 0.015 ng/mL (lower risk).

**Figure 1:**
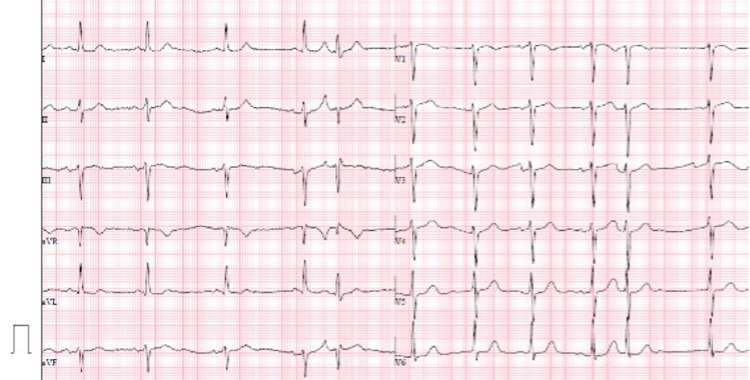
Twelve-lead ECG with atrial fibrillation

**Figure 2:**
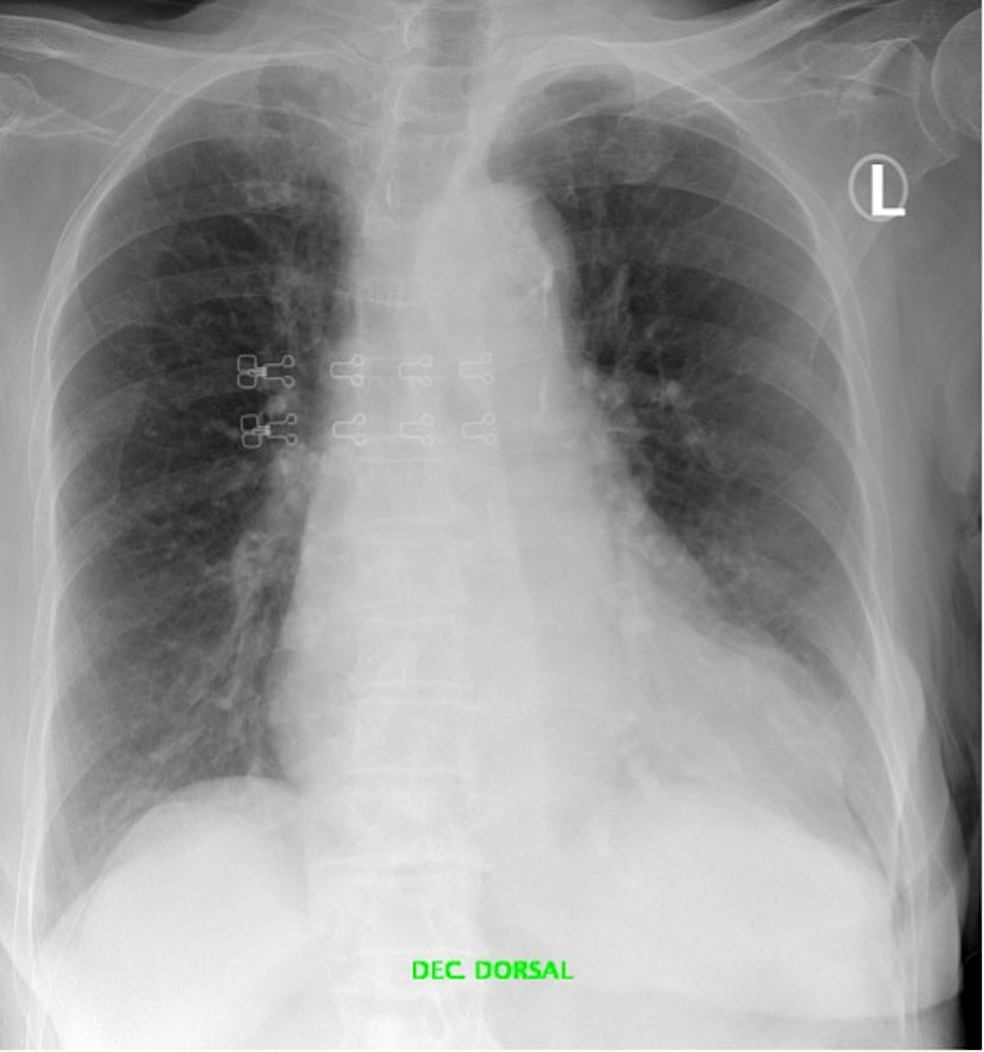
Chest X-ray with mediastinal widening and an enlarged cardiomediastinal silhouette

An initial 12-lead electrocardiogram (ECG) showed atrial fibrillation with controlled ventricular frequency (Figure 1), and there was a new mediastinal widening on the chest X-ray with an enlarged cardiomediastinal silhouette (Figure 2). 

**Figure 3:**
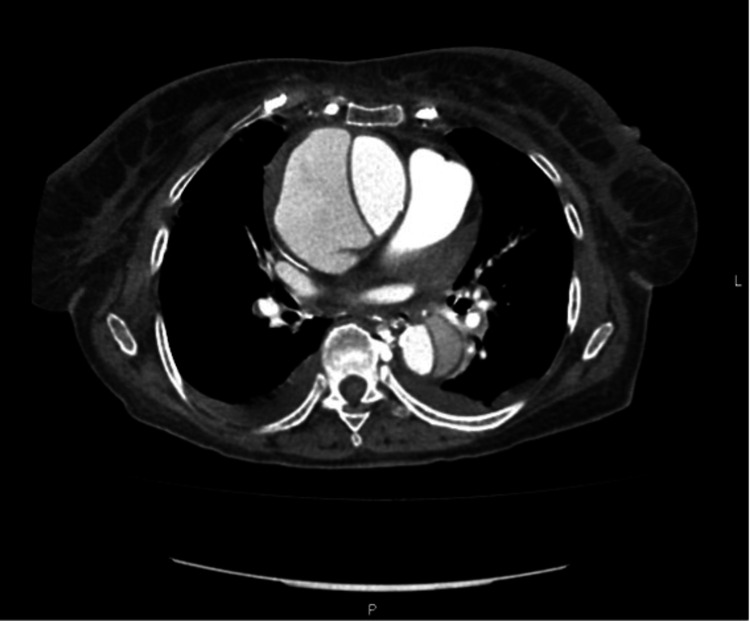
Chest, abdominal, and pelvic CT with a thoracic aortic dissection, DeBakey type I, Stanford type A, extending to the abdominal aorta, the emergence of the celiac trunk in the false lumen and superior mesenteric artery with the emergence in the true lumen

**Figure 4:**
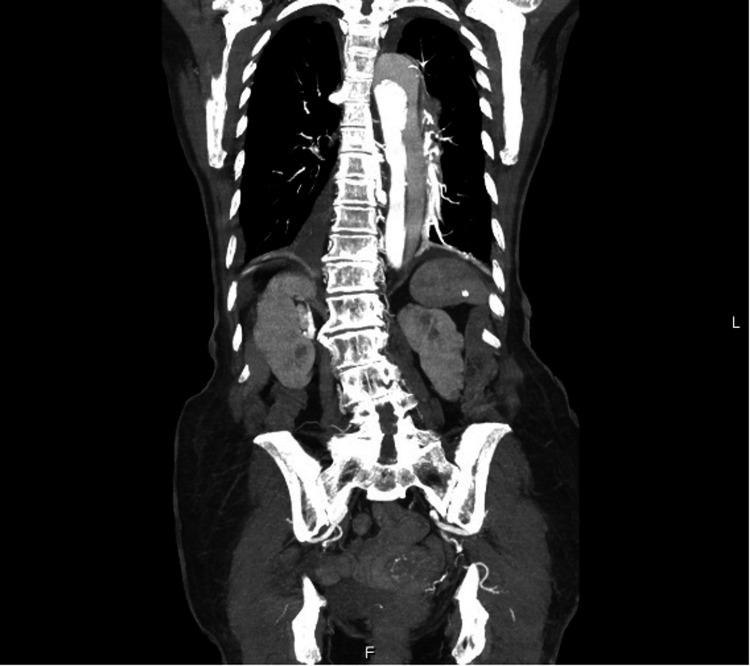
Chest, abdominal, and pelvic CT with a thoracic aortic dissection, DeBakey type I, Stanford type A, extending to the abdominal aorta, the emergence of the celiac trunk in the false lumen and superior mesenteric artery with the emergence in the true lumen

The patient was admitted to the internal medicine department with suspicion of lower airway infection. Blood cultures were collected, and the patient empirically started amoxicillin/clavulanic acid 875/125 mg and clarithromycin 500 mg. Due to the persistence of retrosternal pain, despite the analgesics, a transthoracic echocardiogram was performed and showed a non-dilated left ventricle, moderate concentric hypertrophy, a good global systolic fraction (~65%), without evident changes in segmental kinetics. There was no pericardial effusion but mild aortic regurgitation and mild mitral regurgitation. There was a marked aneurysm of the ascending aorta with a dissection line (visible in the right sternal window). To clarify this finding, a chest, abdominal, and pelvic computed tomography (CT) was requested and revealed a thoracic aortic dissection, DeBakey type I, Stanford type A, extending to the abdominal aorta, the emergence of the celiac trunk in the false lumen and superior mesenteric artery with the emergence in the true lumen (Figures 3-4). There were also signs of active bleeding in the mediastinum with hemomediastinum.

We contacted cardiothoracic surgery, which called the patient’s family to discuss the clinical situation, the risks of not intervening surgically, and the benefits, risks, and probabilities inherent to the surgery. As a result, the patient refused surgery and was transferred to the cardiothoracic surgery department for stabilization and clinical surveillance. The patient had a contraindication to hypocoagulation due to the greater risks than benefits of the ruptured aorta dissection. Close blood pressure monitoring was performed during hospitalization in the cardiothoracic surgery department, and the patient was discharged three days after transfer in a clinically stable state. She returned to the emergency room with 24-hour abdominal pain and prostration about 15 days later. She was admitted to the palliative care service, where she died four days later.

## Discussion

Despite being one of the most common life-threatening aortic disorders, acute aortic dissection represents a rare cardiovascular disease with a fatal outcome. About 1% of untreated patients die per hour after symptoms start [12]. The tragic cases can be related to rupture of the dissection into the pericardium, leading to cardiac tamponade, acute dissection into the aortic valvular annulus precipitating severe aortic regurgitation, obstruction of the coronary artery ostia causing myocardial infarction, and end-organ failure due to abdominal aortic branch vessel obstruction [13-14]. 

Accurate classification of aortic dissection, in terms of the duration of time from its occurrence and anatomical location of the intimal tear, is crucial to define the treatment strategy and prognosis. Considering the time of symptom presentation, acute dissection is defined as within two weeks of symptom start, and chronic dissection is longer than two weeks. The two main anatomical classifications used are the Stanford and DeBakey classifications [15]. The Stanford system classifies type A dissection as one involving the ascending aorta, irrespective of the entry point site of the primary intimal tear. All other dissections starting after the left subclavian artery are classified as type B [15]. The DeBakey classification is based upon the affected aortic segment and its extension. In type 1, the intimal rupture is located in the ascending aorta and propagates to the aortic arch. In type 2, the origin tear derives in and is confined to the ascending aorta, and in type 3, it initiates in the descending aorta and extends distally [16]. The patient in the case presented was diagnosed with an acute aortic dissection involving the ascending aorta, DeBakey type I, Stanford type A.

Contributing factors are varied, and hypertension seems to be the most prevalent risk factor for acute dissection, with a prevalence of 75% of cases [17]. Smoking, dyslipidemia, drugs (cocaine or amphetamines), connective tissue disorders, and deceleration trauma may also contribute to the dissection [17]. The presenting patient had a medical history of arterial hypertension and dyslipidemia.

Patients with acute aortic dissection typically complain of severe persistent chest pain of sudden onset that migrates along with the dissection. In addition, they may be hypotensive and present diminished or unequal peripheral pulses or malperfusion of their lower extremities [18]. Depending on the extent of the dissection, patients may also present mental status or neurological changes (as hemiplegia), pericardial effusion, and involvement of the arch vessels, coronary arteries, and aortic valve [18]. Our patient presented with sudden and persistent retrosternal pain and was hypotensive. The peripheral exam showed regular bilateral peripheral pulses.

Imaging studies have a crucial role in the confirmation of diagnosis, classification of dissection, localization of tears, assessment of extending dissection, and indicators of urgency [17]. A multiplanar CT scan is the diagnostic imaging modality of choice. A chest X-ray may reveal an abnormal aortic contour or widened superior mediastinum. A transesophageal echocardiogram is an excellent diagnostic method for aortic dissection, but it is generally not readily available in the acute setting.

When an aortic dissection is suspected, aggressive measures to limit propagation by lowering blood pressure should start immediately. Treatment requires a reduction of the systolic blood pressure to 100-120 mmHg and pulse pressure to 60-80 beats per min [17]. Intravenous beta-blockers are the first-line treatment, as they reduce the left ventricular ejection force that continues to deteriorate the arterial wall. Urgent surgical intervention is required for all type A dissections, although a patient-centered assessment of the risks and benefits of surgical repair should be made. Emergency cardiac surgery for acute type A dissection in octogenarians’ patients seems controversial since a surgical mortality approach of 50% and 70% of postoperative complications is reported [18]. In the case presented, the patient's family was contacted to discuss the clinical situation, the risks of not intervening surgically, and the benefits, risks, and probabilities inherent to the surgery; they decided against surgical intervention.

## Conclusions

Aortic dissection is a potentially fatal condition whose accurate diagnosis and early and effective treatment are essential for patient survival. Sudden onset of chest pain that patients describe as excruciating, anterior, or interscapular pain is characteristic. Aortic dissection is most commonly misdiagnosed as myocardial infarction or other causes of chest pain such as pulmonary embolization. When an aortic dissection is suspected, aggressive measures to limit propagation by lowering blood pressure should start immediately. Urgent surgical intervention is required for all type A dissections. With this case, the authors intend to emphasize the need to combine the clinical history with signs and symptoms to obtain an early and correct diagnosis, especially when faced with a disease with a potentially fatal prognosis.
